# Latent Class Analysis of Household and Community Adversity Among Pre-Adolescent Youth in the United States

**DOI:** 10.1007/s10578-025-01883-7

**Published:** 2025-07-22

**Authors:** Kristen R. Choi, Erin C. Dunn, W. Scott Comulada, Altaf Saadi

**Affiliations:** 1 School of Nursing, University of California Los Angeles, Los Angeles, CA, USA; 2 Department of Health Policy and Management, Fielding School of Public Health, University of California Los Angeles, Los Angeles, CA, USA; 3 Department of Sociology, College of Liberal Arts, Purdue University, West Lafayette, IN, USA; 4 Department of Psychiatry and Biobehavioral Sciences, David Geffen School of Medicine at UCLA, Los Angeles, CA, USA; 5 Department of Neurology, Massachusetts General Hospital and Harvard Medical School, Boston, MA, USA

**Keywords:** Adversity, Community, Discrimination, Latent class analysis

## Abstract

The purpose of this study was to: 1) examine the co-occurrence of household and community adverse childhood experiences (ACEs) among preadolescent youth using latent class analysis (LCA), and 2) examine the association of ACE latent clusters to clinical-range scores on the Child Behavior Checklist (CBCL). Data came from the baseline and year 1 survey of the Adolescent Brain Cognitive Development (ABCD) Study with 10,915 youth recruited from school-based catchment areas in the United States. We used LCA to examine 6 types of household adversity and 7 types of community adversity, including 4 types of discrimination. We identified 5 latent classes of household/community ACEs. The class with high levels of household and community ACEs together was most strongly associated with clinical-range CBCL scores in adjusted models. Assessing adversity comprehensively may improve identification of youth with elevated risk for behavioral symptoms, who are greatest in need of intervention.

## Introduction

Much existing research on the effects of adverse childhood experiences (ACEs) on health has focused on ten classic ***household*** *ACEs*—defined as maltreatment (sexual abuse, emotional abuse, physical abuse, neglect) and household dysfunction (parent divorce, family member incarceration, parent mental illness or substance misuse) [15]—that can portend a life course of morbidity. Empirical research has shown a dose–response relationship between these ACEs and negative health outcomes, with each additional ACE exposure increasing risk for poor health outcomes later in life by as much as 12-hold higher risk for those with 4 or more ACEs, including behavioral problems and impaired cognitive function across the lifespan [15,18]. One proposed mechanism linking childhood adversity, especially ongoing adversity, to poor health is toxic stress—a prolonged activation of physiological stress response systems that occurs in response to severe, frequent, and prolonged adversity and in the absence of stress-buffering, such as protective relationships with parents or other caregivers [37]. Under the concept of toxic stress, accumulation of adverse experiences and associated stress response increases risk for poor health outcomes across the life course.

Community adversity experienced outside of youth’s homes can also play a negative role in child health and development, particularly as youth age and spend more time outside the home, at school or with peers in the neighborhood [12, 24]. Observational studies suggest youth who grow up in disadvantaged communities with higher poverty, disinvestment in schools, economic resource deprivation, over-policing, and exposure to school or community violence are at greater risk for behavior problems and mental illness [5, 17, 38]. Experimental studies have also shown that receiving vouchers to move to higher-quality neighborhoods can reduce psychological distress and psychiatric service utilization among adolescents [29, 34]. The effects of community disadvantage on youth health may be compounded for racial or ethnic minority youth who are more likely to be born into communities affected by disadvantage and racism [14].

Past research on adversity in neighborhoods or communities has predominantly considered socioeconomic disadvantage and community or school violence, finding associations between exposure to community adversity and impaired cognition as well as structural brain changes associated with risk for mental illness [13, 33]. But fewer studies have examined, together in the same study, the multiple types of adversity that can occur in communities—such as community violence, school violence, community discrimination, and school or neighborhood bullying—or comprehensively examined community adversities alongside household ACEs [10, 11]. Because racial/ethnic minority youth have been found to be at elevated risk for experiencing adversity in their communities, it is especially important to consider racial discrimination and other types of discrimination alongside other forms of community adversity, as these experiences may be related to additive risk when they overlap with other ACEs [26].

There have been prior studies of co-occurring ACEs and other types of trauma, as described in a recent systematic review of latent class analysis studies of ACEs. In this review, person-centered studies of ACEs consistently identify 3–5 distinct latent classes, typically including a low-exposure class and various combinations of higher-risk classes with specific adversity patterns (such as maltreatment, household dysfunction, or poverty-related clusters), with classes characterized by child maltreatment generally most strong associated with poor developmental and behavioral outcomes [43]. Another review of person-centered studies of child maltreatment similarly found evidence for 2–5 classes and that classes characterized by multiple direct abuse types are most strongly associated with negative outcomes [32]. Finally, a review of studies of community violence among adolescents found consistent evidence that direct victimization conferred greater risk for internalizing behaviors than indirect (witnessing) violence [27]. One major gap in these prior studies is including measures of discrimination and focusing on specific developmental epochs to understand patterns of ACE exposure across the life course. For example, adolescence is a significant developmental period in the life course when ACEs occurring during childhood may start or continue to affect behavioral health,ACE exposure may also continue to persist into adolescence. As children enter adolescence and begin spending more time outside of their homes, they may be at increased risk for exposure to community adversity, making it important to understand how community adversities co-occur with household ACEs to affect early adolescent behavioral health.

Despite this knowledge gap, community forms of adversity, like bullying, bullying, neighborhood violence, and discrimination, are now being included in some screening tools used to assess ACEs in clinical care [1, 39]. We therefore leveraged the inclusion of these kinds of variables in one larger-scale national study to: 1) examine the co-occurrence of household and community ACEs among preadolescent youth using latent class analysis (LCA), and 2) examine the association of ACE latent classes to child behavioral problems. In line with prior research, we used LCA to group individual ACE items for interpretability and preserve item variability in the analyses [3, 6, 9]. We conducted two sets of analyses for each aim; first, using household ACEs only and second, adding community ACEs to assess for changes in ACE clustering. LCA allows for identification of clusters of ACEs, which is an advancement from representing ACEs as counts as they have traditionally been measured in past research [7]. Understanding the role of ACEs in the adolescent developmental period has potential to inform the development of interventions for identification and response to high-risk patterns of ACEs.

## Methods

### Study Design and Data

We analyzed baseline and year 1 data from the Adolescent Brain Cognitive Development (ABCD) Study (5.0 ABCD data release) [41]. The ABCD Study is a national, prospective study of youth brain development and health initiated in 2015. The study is prospectively following a cohort of approximately 12,000 pre-adolescent youth ages 9–10 years for 10 years, with current data collection ongoing. Household ACEs were most comprehensively measured at baseline, while most community ACEs were first measured at year 1 leading to the inclusion of year 1 data for these measures only; all other measures were assessed at baseline. Youth in the ABCD sample were recruited from 21 school-based catchment areas, to be representative of the US population [16]. Additional details about the ABCD study data and methods are reported elsewhere. The Institutional Review Board (IRB) at [blinded for review] determined the study exempt from review as this is a secondary analysis of de-identified data.

### Sample

We included all youth in the ABCD cohort who had baseline and year 1 data available. There were 11,878 in the baseline sample and 11,220 youth remaining at year 1 follow-up. Of youth with baseline and year 1 data available, there were 306 youth (3% of the one-year sample) missing data on one or more study measures and these youth were excluded from the analytic sample for a final analytic N of 10,915. Black and Hispanic youth were over-represented among those excluded for missing data (P < 0.01).

### Measures

#### Household ACEs

Consistent with prior studies [21], eight of the ten ACEs were derived from baseline items in the ABCD study (physical abuse, sexual abuse, emotional neglect, domestic violence, parent substance misuse, parent mental illness, parent separation/divorce, and parent legal involvement). All ACE measures were constructed using parent-report items, except for emotional neglect, which was youth-report. The ABCD study does not include measures of emotional abuse or physical neglect. We assessed ACEs as binary (exposed versus unexposed) variables as well as an overall count using measures recommended for capturing ACEs in the ABCD study [21].

#### Community ACEs

We assessed seven community ACEs including discrimination across four domains (race/ethnicity, nationality, weight/body size, gender identity), bullying, and witnessing and experiencing community violence. The ABCD perceived discrimination measure assesses the presence or absence of four types of discrimination using items adapted from the 2006 Boston Youth Survey [19]. The youth-report items asked about past 12-month perceived discrimination in four domains: racial/ethnic discrimination, nationality/country of origin discrimination, gender identity discrimination, and weight/body type discrimination (e.g., “Have you felt discriminated against because of your weight?”). We examined each discrimination item as a binary variable. We also examined the presence of bullying (“Does your child have any problems with bullying at school or in your neighborhood?”), derived from a mental health diagnostic interview, and witnessing and experiencing violence from the parent-report life events measure (responses to “saw crime or accident?” and “was a victim of crime/violence/assault?”, respectively).

#### Behavioral health outcomes

We used the Child Behavior Checklist (CBCL), a 113-item measure of emotional and behavioral problems among youth, scored on a three-point Likert scale of problem frequency [2]. The CBCL was completed by parents/care-givers at baseline and has two broadband scales for internalizing and externalizing behavior problems. Internalizing behaviors reflect withdrawn mood disturbances, including depression and anxiety, while externalizing behaviors entail aggression, attentional, oppositional symptoms. Broadband scores are age-normed into T scores with a mean of 50 and standard deviation of 10. Scores of 65–69 are considered borderline for a clinical-range behavioral problem, while scores of 70 or higher are indicative of a clinical-range problem. We examined CBCL T scores overall, plus internalizing and externalizing broadband T scores.

#### Demographics

Youth demographic factors were examined to characterize the sample and compare differences in ACE exposure. The following demographic factors, reported by parents, were examined: youth gender (boy, girl, non-binary); race/ethnicity (Asian, Black, Hispanic, Multiracial, Native American Indian/Alaskan Native, Other, White); parent marital/partner status; and parent educational attainment as an indicator of family economic stability.

### Analysis

Data were analyzed using R, version 4.3.1 [30]. We applied LCA to identify overlap in household and community ACEs. LCA is used to identify unobserved heterogeneity (i.e., an unobserved latent “class” variable) in a population [28, 35]. LCA is a person-based approach to data analysis that groups similar individuals based on their responses to a set of observed input variables, generating a latent class variable for group membership. In our analysis, ACE latent classes were enumerated by fitting a series of latent class models with the same observed variables, starting with two classes and adding one additional class at a time to determine the optimal number of classes. The models were compared to confirm the best-fit model that was both statistically supported and conceptually interpretable, using Bayesian Information Criterion (BIC), Akaike Information Criterion (AIC), Lo-Mendell-Rubin Likelihood Ratio Tests, and Chi-Square tests. Of these, we prioritized BIC values because they tend to favor more parsimonious models [28].

We conducted two LCAs following this process to understand the overlap of household ACEs at baseline and community ACEs reported one year later: 1) an LCA of household ACEs only (eight input variables) and 2) an LCA of an expanded set of ACEs including community ACEs (13 input variables). These two LCAs were conducted to assess whether there were changes in class characteristics and ACE clustering patterns when community ACEs were included. In a subsequent LCA, the sexual abuse, physical abuse, and emotional neglect items were combined into a single maltreatment indicator for a total of six household and seven community ACEs, as these maltreatment items had very low frequency (0.7 to 1.5%).

Following class identification, we assigned each ABCD participant to their most likely latent class based on class membership probabilities (latent class prevalence) and assessed item response probabilities—that is, the probability of an indicator being present among members of a given latent class—in line graphs to compare ACE-related differences among classes [23]. We used chi-square tests, frequencies, and descriptive statistics to further characterize each latent class by race/ethnicity, gender, household and community ACEs, and clinical-range CBCL T scores. Then, we estimated linear mixed effects models to assess which ACE latent classes significantly associated with clinical-range CBCL T scores (total, internalizing, externalizing), accounting for 1) clustering within families to address similar household experience by siblings and 2) clustering within study site using the ‘gamm4’ R package as recommended for population-based analysis with this dataset [20, 44]. Models also adjusted for demographic covariates (child gender and race, parent marital status and educational attainment). To increase the rigor and reproducibility of our results, all steps of analysis underwent an independent code review to identify and resolve discrepancies [40].

## Results

### Sample description

The sample was 47.4% girls (n = 5179), 53.9% White (n = 5878), 15.7% Hispanic (n = 1709), and 14.9% Black/African American (n = 1626) (Table 1). Overall, 46.1% of youth (n = 5036) reported at least one household ACE and 12% (n = 1310) had at least one community ACE. Twelve percent of youth reported any discrimination, with weight/body size discrimination reported most frequently (n = 642, 5.9%) among discrimination types. Three percent of the sample (n = 301, approximately 22.9% of all youth who reported discrimination) experienced more than one type of discrimination. Discrimination based on nationality/country of origin was least frequent (n = 171, 1.6%). Fifteen percent of the sample reported bullying (n = 1662); 7.1% reported witnessing community violence (n = 772); and 1.4% reported crime victimization (n = 154). Three percent of the sample (n = 351) had a clinical-range CBCL T score.

### Household ACE LCAs (LCA 1)

**Fig. 1:**
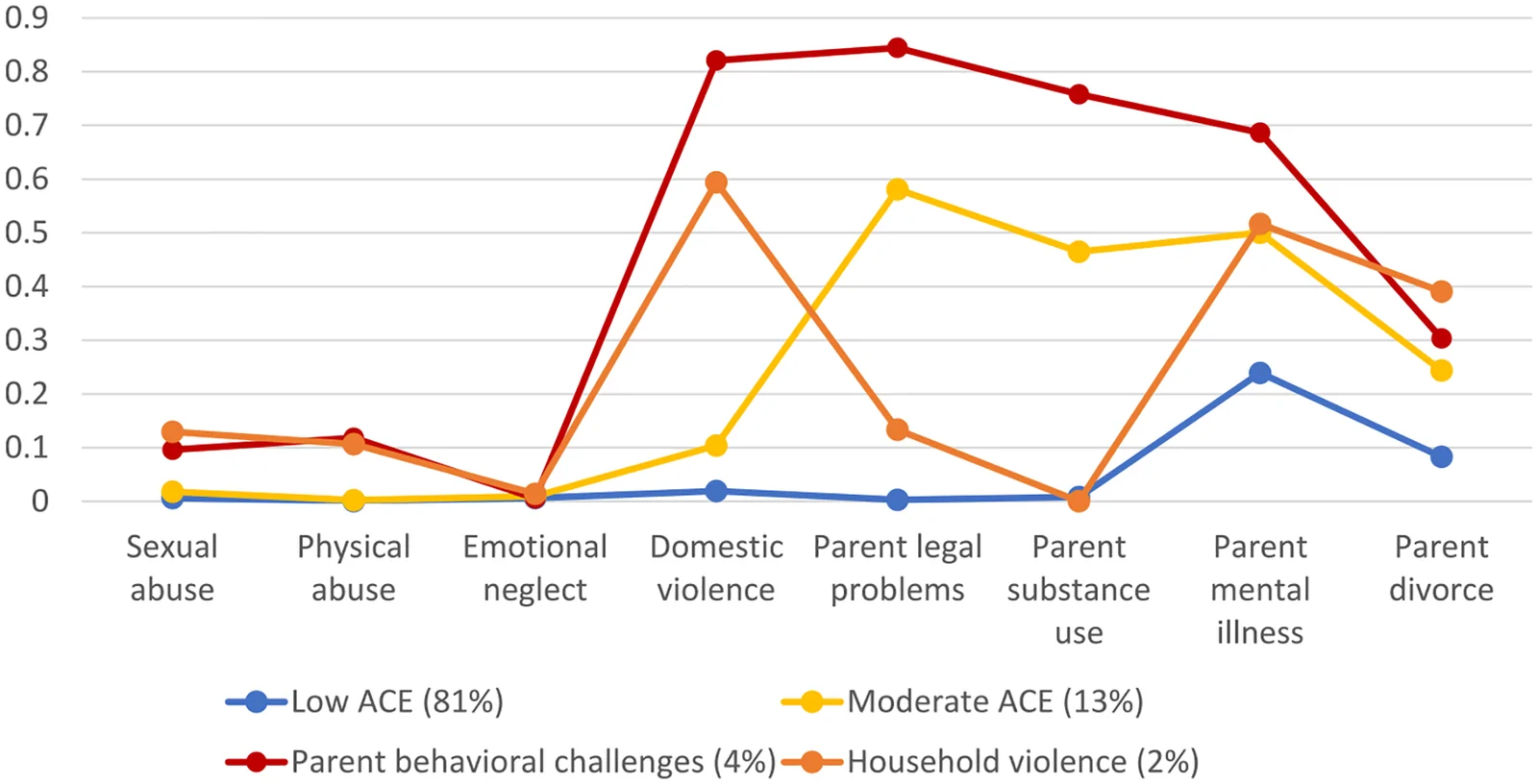
Item response probabilities for a 4-group latent class analysis of adverse childhood experiences in households. Item response probabilities derived in a latent class analysis (LCA) of 8 household adverse childhood experiences (ACEs) in a sample of 10,915 pre-adolescent youth in the United States. A 4-class solution was selected based on model fit statistics

In the first LCA using eight household ACEs as input variables, we tested 2, 3, 4, and 5-class solutions and found that a 4-class model was the best fit for data (Fig. 1, Table 2). There was a low-ACE class (80.8% of the sample, n = 8824) with low probability of having household ACEs of any kind, plus a moderate-ACE class that had a similar pattern of item response probabilities but with higher probabilities for ACE items compared with the low-ACE class (13.2% of the sample, n = 1446). The next class was characterized by risk relating to parent- behavioral challenges (e.g., parent mental illness, substance misuse), but not maltreatment (3.5% of the sample, n = 385). The last class with 2.4% of the sample (n = 260) comprised youth with high probability of being exposed to household violence including multiple maltreatment and parent-related ACEs, particularly exposure to domestic violence.

### Expanded ACE LCA (LCA 2)

**Fig. 2:**
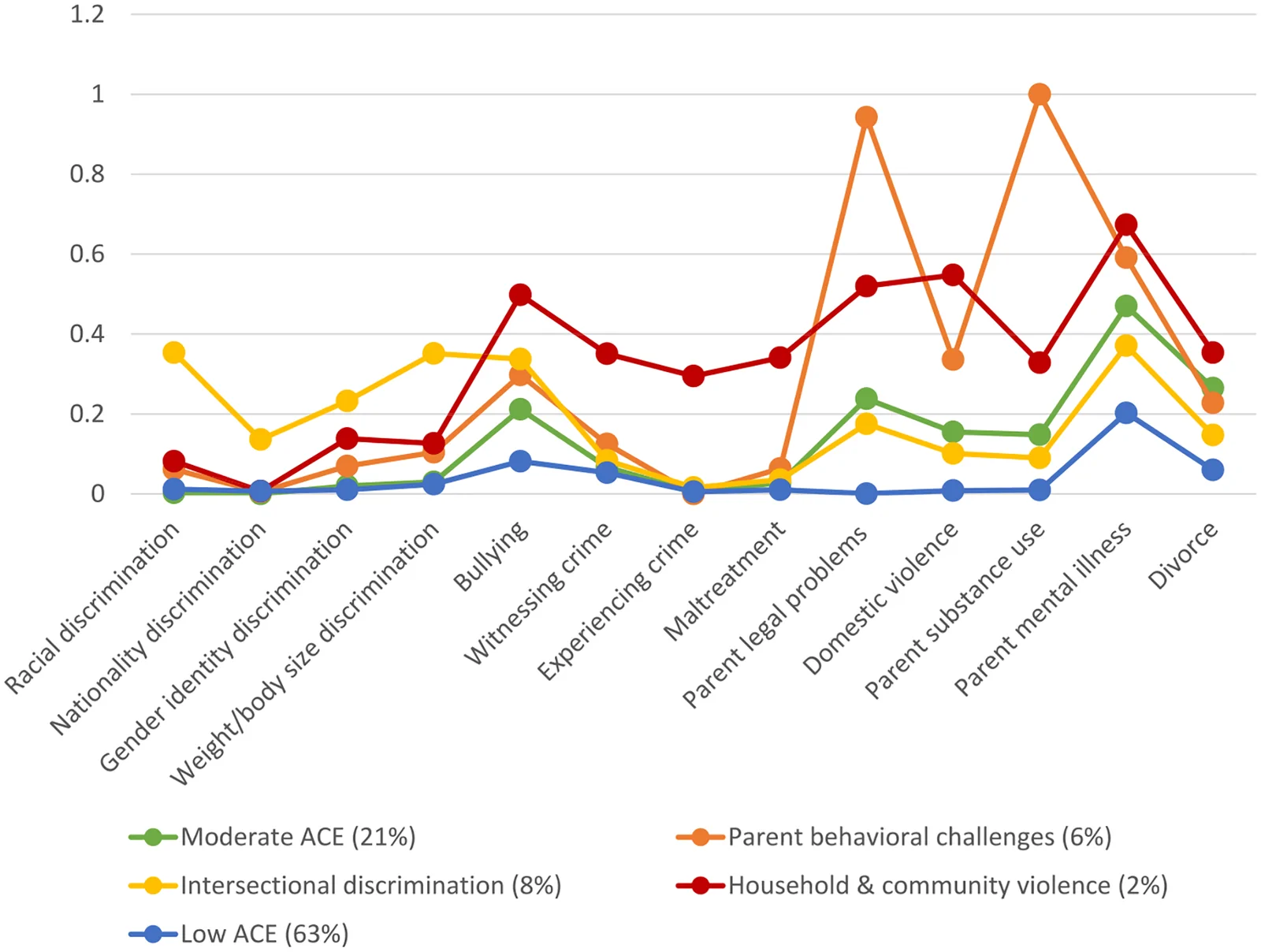
Item response probabilities for a 5-group latent class analysis of adverse childhood experiences in households and communities. Item response probabilities derived in a latent class analysis (LCA) of 6 household adverse childhood experiences (ACEs) and 7 other community ACEs in a sample of 10,915 pre-adolescent youth in the United States. A 5-class solution was selected based on model fit statistics

In the second LCA, we used all household and community ACEs (13 total) as input variables and tested 2, 3, 4, 5, and 6-class solutions (Fig. 2,Table 2). We found that a 5-class model was favored by BIC and therefore we selected it as the best fit for the data. A comparison of the five latent classes is shown in Table 1 by demographic and ACE frequency characteristics. There was a large low-ACE class containing 72.4% of the sample (n = 7902), plus four smaller classes with differing patterns of ACEs. The first of these classes comprised youth who were most prominently affected by ACEs, with high probability of both household and community violence experiences (2.0%, n = 213). This class had high levels of exposure to three or more household ACEs among classes (17.4% of class members compared to 8.2% in the sample as a whole, P < 0.01) and high levels of clinical-range CBCL scores (14.1% of class members compared to 3.2% of overall sample, P < 0.01), as shown in Table 1. The second ACE class (5.9% of the sample, n = 640) was characterized by risk for multiple forms of intersectional discrimination, with higher probably of experiencing community adversities compared with other classes. Eighteen percent of these class members had three or more community ACEs, compared with 2% of the sample as a whole (P < 0.01). This class had over-representation of Black (27.3% of class versus 14.9% of overall sample) and Hispanic (18% of class versus 15.6% of overall sample) (P < 0.01) youth compared with the sample as a whole and the lowest percentage of White youth (37.3% of class versus 53.9% of overall sample). The third ACE class had moderate ACE levels compared with the low-ACE class (13.8%, n = 1505). There was greater overall risk for ACEs in other classes, and in the moderate ACE class there were not any particular ACEs that had especially high or low probabilities that gave this class a unique ACE risk profile. Finally, the last class was similarly characterized by elevated risk for parent behavioral challenges such as legal problems, substance misuse, and mental illness compared to the lowest-ACE class, but less risk than the highest-ACE class (5.6%, n = 654). We did not observe any differences in class membership by gender.

There was high overlap between the low-ACE classes in LCA 1 and LCA 2, with 95.3% overlap. In LCA 2 with community ACEs added, there was evidence for greater differentiation of ACE subtypes as youth who were classified as low-ACE in LCA 1 were classified into more specific ACE subtypes in LCA 2. Specifically, 463 youth were re-classified from the low-ACE class in LCA 1 to the intersectional discrimination class in LCA 2; 639 were re-classified into the moderate ACE class; and 122 were re-classified into the parent behavioral challenges class.

### Association of ACE latent classes to behavioral symptoms

In the final model (Table 3), we found that membership in all of the ACE latent classes was associated with higher overall CBCL T scores (with reference to the low ACE class). The strongest association was for the household and community violence class, which was associated with 8 additional overall CBCL T score points relative to the low ACE class (SE = 0.72, P < 0.001). Magnitudes of association were similar when examining internalizing and externalizing broadband scores; that is, membership in the household and community violence class was most strongly associated with broadband T scores (internalizing β = 6.3, SE = 0.7, P < 0.001; externalizing β = 6.7 SE = 0.7, < 0.001).

## Discussion

This study explored the overlap of household and community adversity in a national sample of pre-adolescents and offers two main findings that contribute to existing literature. First, using LCA, we identified which household and community ACEs co-occurred with one another, finding several distinct patterns of household/community ACE overlap. Second, we identified that the latent class characterized by the highest levels of household and community ACEs together was also the class with the highest levels of clinical-range CBCL scores and the class most strongly associated with higher CBCL scores in adjusted regression models. Our analysis suggests conceptualizing child adversity broadly, beyond household experiences alone, may improve identification of youth with high-ACE exposure and who may be at elevated risk for behavioral symptoms and thus in need of intervention.

One notable finding from this study was identification of an ACE pattern characterized by discrimination. The intersectional discrimination class (5.9% of the sample) was characterized by high probability of experiencing multiple forms of discrimination and other community adversities, with significant overrepresentation of Black and Hispanic youth relative to the sample as a whole. While this class had elevated CBCL scores compared to the low-ACE class, these elevations were less pronounced than for the household and community violence class, which aligns with prior research suggesting that direct threats to caregiver relationships may have particularly detrimental effects on child behavioral health [8]. This finding raises important questions for future research about potential protective factors that might buffer the impact of discrimination on behavioral symptoms, such as having strong sense of ethnic identity. Future research should explore whether similar patterns of discrimination extend across developmental periods and examine potential mechanisms that might explain the differential impact of discrimination compared to other forms of community and household adversity.

Our study extends prior LCA investigations of trauma, adversity, and the role of exposure latent classes in shaping child behavioral health and development, particularly by examining community adversities with disaggregation of experiences of discrimination and community violence. Previous studies examining patterns of co-occurring ACEs have found three to five distinct exposure patterns, ranging from low-exposure groups to higher-risk clusters that may center around maltreatment or household dysfunction exposures [32, 43]. Classes involving multiple forms of abuse and direct rather than indirect abuse have had the strongest associations with negative outcomes [27, 32]. Our study had parallel findings. We found that youth with patterns of co-occurring household and community adversity had the highest risk for behavioral symptoms, which has been found previously with other large samples of youth, such as the National Survey of Children’s Health and National Longitudinal Study of Adolescent and Adult Health [9, 25]. Several studies have examined co-occurring household ACEs among foster care-involved youth and children with a known history of maltreatment [31], including one study that stratified children by developmental stage from infancy to adolescence and assess trauma classes within stages [4], and had similar findings around co-occurring community adversities. Our findings provide further evidence that types of adversity, in addition to frequency of adversity which has been found to be a risk factor in prior studies (Felitti et al., 1998b), shape behavioral health risk, with adversities that disrupt or threaten caregiver relationships consistently associated with the greatest risk.

In our study, pre-adolescent youth with the highest probability of co-occurring household and community ACEs also had the highest levels of clinical-range CBCL scores. Several other LCAs involving ACEs have found a relationship between high-ACE latent classes and risk for substance use, depression, and anxiety in adult samples [22, 36]. However, it is important to note that in this pre-adolescent sample, certain ACEs were reported with relatively low frequency (e.g., maltreatment). As mental illness prevalence increases during adolescence and early adulthood, there is opportunity to assess mental health and substance use trajectories associated with ACE latent classes as the ABCD cohort ages, identifying the specific role of ACEs pre-adolescence.

Our study had strengths and limitations. We used a large, national sample of pre-adolescent youth, which is a strength relative to past LCA studies that have focused on highly trauma-exposed youth such as those interfacing with child welfare systems. In this community sample of youth, we found similar results to clinical and high-risk studies. Even though the frequency of adversity was lower in our sample, which may be due to the community rather than clinical nature of the sample, age of participants, or assessment of maltreatment in a clinical interview, comparable findings across samples supports the validity of ACE latent classes. We also measured multiple household and community ACEs, including distinct forms of discrimination, to understand ACE overlap. However, we relied on a mix of parent-and youth-report measures, as measures from both reporters were not available for most ACE items in the ABCD; there is evidence for disagreement between parents and children in reporting exposure to trauma [42]. We lacked simultaneous measurement of all adversity variables, as community adversities were first measured at Year 1 follow up while household ACEs were measured at baseline. Although this timing allows for identification of which community ACE patterns follow pre-adolescent household ACE patterns, it would have been ideal to measure all items simultaneously and with direct ACE measures, as well as to include a broader set of potentially adverse experiences in communities. Further, the analytic sample had a smaller percentage of Hispanic and Asian youth than US estimates, and thus there may be underestimation of the impact of certain types of discrimination (i.e., racial and nationality-based discrimination) necessitating further and expanded studies on discrimination among youth of color and immigrant youth. Our study did not account for detailed caregiver factors that might have buffered toxic stress associated with adversity, or other potential buffers and protective factors. These factors should be studied in the context of multiple ACE types in future research.

Our findings about different ACE latent classes can inform the targeted development and implementation of interventions. For example, we also found one class with three or more community ACEs and where Black and Hispanic youth were over-represented, that supports prior research about the vulnerability of minoritized youth who experience structural disadvantage. This suggests the need for both interventions that are tailored towards this population, but also multi-level interventions that address social and public policies that perpetuate structural harms for these groups. Similarly, we found a latent class that involved primarily those exposed to domestic violence, suggesting potentially unique interventional needs for this population. Considering different grouping of adversities can help to make interventions more precise and attuned to youth and family needs.

In summary, results from this study show various forms of adversity, both in the household and the community, can influence a child’s behavioral well-being. As such, it is important to not only consider traditional household ACEs but also to recognize the importance of community-based adversities, such as discrimination, bullying, and exposure to community violence [45]. Future research and clinical care efforts to address ACEs may incorporate attention to community-based adversities and corresponding stress buffers, especially as it relates to developing tailored interventions for those at the highest risk for behavioral problems during pre-adolescence.

## Figures and Tables

**Table 1:** Comparison of LCA 2 ACE latent classes

	Overall sample (N = 10,915)		Low ACE latent class (n = 7902)		Household & community violence latent class (n = 213)		Intersectional discrimination latent class (n = 640)		Moderate ACE latent class (n = 1505)		Parent behavioral challenges latent class (n = 654)		P
	N	%	N	%	N	%	N	%	N	%	N	%	
**Race/ethnicity**													<.001
Asian	206	1.9%	178	2.3%	–	–	12	1.9%	13	0.9%	–	–	
Black	1626	14.9%	990	12.5%	37	17.4%	175	27.3%	296	19.7%	128	19.6%	
Hispanic	1709	15.7%	1229	15.6%	24	11.3%	115	18.0%	248	16.5%	93	14.2%	
Multiple	1357	12.4%	891	11.3%	41	19.2%	88	13.8%	217	14.4%	119	18.2%	
Other	137	1.3%	91	1.2%	–	–	11	1.7%	22	1.5%	–	–	
White	5878	53.9%	4523	57.2%	108	50.7%	239	37.3%	707	47.0%	301	46.0%	
**Gender**													0.2
Girls	5719	52.4%	4172	52.8%	124	58.2%	329	51.4%	755	50.2%	339	51.8%	
Boys	5179	47.4%	3719	47.1%	88	41.3%	309	48.3%	747	49.6%	315	48.2%	
**Household ACEs**													<.001
0	5828	53.4%	6177	78.2%	44	20.7%	111	17.3%	1029	68.4%	378	57.8%	
1	3061	28.0%	1541	19.5%	66	31.0%	128	20.0%	418	27.8%	195	29.8%	
2	1130	10.4%	145	1.8%	65	30.5%	287	44.8%	53	3.5%	65	9.9%	
3 or more	896	8.2%	39	0.5%	37	17.4%	114	17.8%	–	–	16	2.4%	
**Community ACEs**													<.001
0	7739	70.9%	6177	78.2%	44	20.7%	111	17.3%	1029	68.4%	378	57.8%	
1	2349	21.5%	1541	19.5%	66	31.0%	128	20.0%	418	27.8%	195	29.8%	
2	615	5.6%	145	1.8%	65	30.5%	287	44.8%	53	3.5%	65	9.9%	
3 or more	211	1.9%	39	0.5%	37	17.4%	114	17.8%	–	–	16	2.4%	
CBCLT score ≥70	351	3.2%	165	2.1%	30	14.1%	37	5.8%	64	4.3%	55	8.4%	<.001
**LCA1 Class Membership**													<.001
Low ACE	8824	80.8%	7534	95.3%	65	30.5%	463	72.3%	639	42.5%	122	18.7%	
Household violence	260	2.4%	65	0.8%	37	17.4%	24	3.8%	132	8.8%	2	0.3%	
Moderate ACE	1446	13.2%	240	3.0%	45	21.1%	142	22.2%	651	43.3%	368	56.3%	
Parent behavioral challenges	385	3.5%	63	0.8%	66	31.0%	11	1.7%	83	5.5%	162	24.8%	

Comparison of final latent classes based on two latent class analyses (LCAs) conducted in a sample of 10,915 pre-adolescent youth in the United States, first using 6 household adverse childhood experiences (ACEs) as inputs (LCA 1) and then second, adding 7 community ACEs for a total of 13 inputs (LCA 2). A five-class solution in LCA 2 was selected as the final model, with the five groups shown above in columns. CBCL = child behavior checklist. Cell counts < 20 were suppressed. Children who identified their gender as other than boy/girl are not shown due to small cell counts

**Table 2:** Model fit statistics for two latent class analyses of adverse childhood experiences

	AIC	BIC	Likelihood ratio test	Chi-square test
**LCA 1 (Household ACEs only)**				
2 Classes	43,523.43	43,647.49	434.4979	1353.504
3 Classes	43,396.27	43,586.02	289.3461	510.602
4 Classes	43,318.27	43,573.7	193.345	296.2782
5 Classes	43,296.81	43,617.92	153.8868	213.1973
**LCA 2 (Household ACEs plus community adversity)**				
2 Classes	72,326.11	72,523.15	2851.946	23,732.65
3 Classes	71,778.74	72,077.95	2276.576	18,709.3
4 Classes	71,611.53	72,012.91	2081.371	8602.112
5 Classes	71,504.04	72,007.59	1945.881	8425.148
6 Classes	71,436.24	72,041.96	1850.077	5542.038

Model fit statistics for two latent class analyses (LCAs) conducted in a sample of 10,915 pre-adolescent youth in the United States, first using 6 household adverse childhood experiences (ACEs) as inputs (LCA 1) and then second, adding 7 community adversities for a total of 13 inputs (LCA 2). AIC = Akaike information criterion; BIC = Bayesian information criterion

**Table 3:** Association of household and community ACE latent classes to behavioral symptoms

	Total CBCL T score			Internalizing T score			Externalizing T score		
ACE latent class (reference: Low ACE)	β	SE	P	β	SE	P	β	SE	P
Household & community violence	7.94	0.72	<.001	6.31	0.70	<.001	6.73	0.67	<.001
Intersectional discrimination	3.65	0.42	<.001	2.58	0.41	<.001	2.99	0.39	<.001
Moderate ACE	2.62	0.30	<.001	2.24	0.29	<.001	2.52	0.28	<.001
Parent behavioral challenges	4.53	0.42	<.001	3.45	0.42	<.001	4.28	0.39	<.001

Linear mixed effects modes estimating Child Behavior Checklist (CBCL) T scores from latent classes of adverse childhood experiences (ACEs, 5 classes total total). Estimates are adjusted for child gender and race, parent marital status and educational attainment, and incorporate household plus site clustering

## Data Availability

Data used in this study may be requested from the NIMH data archive: https://nda.nih.gov/abcd.
